# Evolution and Single‐Droplet Analysis of Fuel‐Driven Compartments by Droplet‐Based Microfluidics

**DOI:** 10.1002/anie.202203928

**Published:** 2022-06-24

**Authors:** Alexander M. Bergmann, Carsten Donau, Fabian Späth, Kevin Jahnke, Kerstin Göpfrich, Job Boekhoven

**Affiliations:** ^1^ Department of Chemistry Technical University of Munich Lichtenbergstrasse 4 85748 Garching Germany; ^2^ Biophysical Engineering Group Max Planck Institute for Medical Research Jahnstraße 29 69120 Heidelberg Germany; ^3^ Department of Physics and Astronomy Heidelberg University 69120 Heidelberg Germany

**Keywords:** Artificial Organelles, Droplet-Based Microfluidics, Nonequilibrium Processes, Phase Transitions

## Abstract

Active droplets are a great model for membraneless organelles. However, the analysis of these systems remains challenging and is often limited due to the short timescales of their kinetics. We used droplet‐based microfluidics to encapsulate a fuel‐driven cycle that drives phase separation into coacervate‐based droplets to overcome this challenge. This approach enables the analysis of every coacervate‐based droplet in the reaction container throughout its lifetime. We discovered that the fuel concentration dictates the formation of the coacervate‐based droplets and their properties. We observed that coacervate‐based droplets grow through fusion, decay simultaneously independent of their volume, and shrinkage rate scales with their initial volume. This method helps to further understand the regulation of membraneless organelles, and we believe the analysis of individual coacervate‐based droplets enables future selection‐ or evolution‐based studies.

## Graphical abstract

Droplet‐based microfluidics were used to encapsulate a fuel‐driven cycle that drives phase separation into coacervate‐based droplets. This approach enables the analysis of every coacervate‐based droplet in the reaction container throughout its lifetime. The nucleation, growth, and dissolution of active coacervate‐based droplets were investigated with this setup.

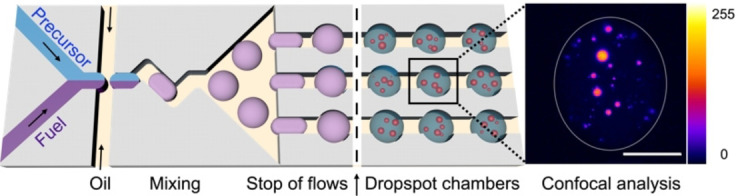

Liquid–liquid phase separation (LLPS) as an underlying mechanism for forming membraneless organelles is attracting increasing attention due to its role in regulating intracellular processes. It is involved in the promotion^[1]^ and suppression^[2]^ of gene transcription, signal transduction,^[3]^ or stress response.^[4]^ These organelles have regulatory properties, but their formation and dissolution are also regulated through chemical reactions such as methylation^[5]^ or phosphorylation.^[6]^ There is increasing evidence that misregulation of these organelles leads to diseases like amyotrophic lateral sclerosis.^[7]^ To better study the regulation of the formation and dissolution of these membraneless organelles, artificial LLPS systems have been developed. Reversible LLPS based on the principle of complex coacervation has been achieved through changes in pH,^[8]^ temperature,^[9]^ salt concentration, or in response to UV light.^[10]^ In these cases, the formation and dissolution of coacervate‐based droplets are regulated by changes in their environment, shifting the system from one equilibrium position to another. Another approach is to regulate the formation and dissolution of these coacervate‐based droplets by chemical reactions, as it is also observed for membraneless organelles in cells. This can be done through reversible phosphorylation^[11]^ or methylation^[12]^ with enzymes or entirely artificial through reversible anhydride formation with carbodiimides.^[13]^ Regulation by chemical reactions also opens a pathway for new emergent properties of these coacervate‐based droplets. It has been predicted that life‐like behavior like size control or self‐division is possible for these active droplets.^[14]^

However, microscopy analysis of active coacervate‐based droplets has been limited. This limitation is partly because only a small fraction of the total reaction solution can be imaged. On the one hand, this leads to a bias depending on which part of the solution is imaged, e.g., imaging close or far away from the top or bottom coverslip can change the average size of the observed coacervate‐based droplets. On the other hand, droplets cannot be tracked over their entire lifetime. Instead, a snapshot of some of the droplets is obtained. Finally, the time of mixing and imaging is relatively slow, as droplets can already form and grow in the first tens of seconds after mixing. These limitations are challenging for further developing active droplets and studying their behavior within a population.

Microfluidic technology is emerging as an essential tool in the analysis of LLPS systems,^[15]^ and especially droplet‐based microfluidic techniques are valuable to overcome these limitations for the analysis of LLPS systems. It has been used to encapsulate coacervate‐based droplets into cell‐sized compartments like stabilized water‐in‐oil droplets[^9b^, ^16^] and lipid vesicles.[^8^, ^17^] This enables high‐throughput screening of coacervate‐based droplets regarding their phase transition behavior^[18]^ or viscosity,^[19]^ and the measurement of partitioning coefficients of different molecules or the influence of coacervate‐based droplets on reaction rates.^[20]^ Encapsulation into liposomes is an efficient way of studying pH‐responsive coacervate‐based droplets, i.e., understanding coacervate‐membrane interactions^[8b]^ and the activation of dormant enzymatic reactions by the formation of coacervate‐based droplets.^[8a]^ Less attention has been paid to studying dynamic properties like nucleation[^16^, ^21^] or dissolution of active coacervate‐based droplets. In particular, fuel‐driven, active droplets are an exciting target for these analyses as their properties are highly dynamic and switch from nucleation, growth, and collapse through dissolution and self‐division in minutes.

Therefore, this work aims to introduce a method that allows us to analyze the behavior of every fuel‐driven active droplet formed throughout its lifetime in an experiment. Thus, we use a microfluidic setup to analyze a fuel‐driven LLPS system in a confined volume. This allows simultaneous analysis of every droplet in the reaction volume immediately after the start of the reaction cycle and tracking and analysis of the behavior of individual droplets from their nucleation to dissolution.

In this work, we use a fuel‐driven reaction cycle coupled with the formation of coacervate‐based droplets that we recently introduced.^[13]^ The system consists of a precursor peptide Ac‐F(RG)_3_D‐OH and either the polyanion polystyrene sulfonate (pSS) or polyuridylic acid (pU). The aspartic acid moiety of the peptide precursor can be activated to its corresponding anhydride through the reaction with 1‐ethyl‐3‐(3‐dimethylaminopropyl) carbodiimide (EDC) as the fuel (Figure 1a). The activated peptide (i.e., the anhydride) rapidly hydrolyzes back to the initial precursor peptide. The constant fuel‐driven activation and deactivation continue until all fuel is depleted. Thus, the addition of fuel results in a population of droplets regulated by the reaction kinetics of activation and deactivation when the reaction is coupled to droplet formation. To do so, the peptide was designed such that activation leads to the negation of two negative charges and converts the overall charge of the peptide from +1 to +3. In its activated state, the affinity of the peptide for the polyanion increases, and, when sufficient peptide has been activated, phase separation through complex coacervation can occur (Figure 1b). However, in the droplet, the peptide can be deactivated through hydrolysis, after which it leaves the droplet. Thus, the droplets are governed by constant in‐ and out‐flux of droplet materials which is regulated through the kinetics of activation and deactivation. Coacervate‐based droplets are thus present in the system as long as a sufficient product concentration can be maintained through fuel consumption.

**Figure 1:**
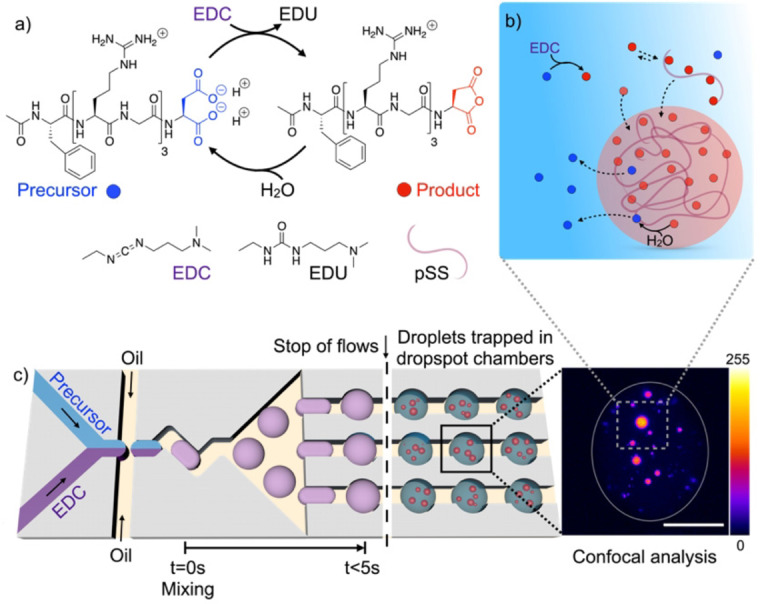
Schematic representation of the reaction cycle and the microfluidic setup. a) Reaction cycle of the precursor Ac‐F(RG)_3_D‐OH with EDC. EDC converts the +1 charged precursor to the +3 charged anhydride product. The product can then hydrolyze back to the precursor. This cycle continues until EDC is depleted. b) Formation and dissolution of coacervates in a confined volume depending on the reaction cycle. The product (red) forms coacervates with polystyrene sulfonate (pSS). Once the product is hydrolyzed back to the precursor (blue), the coacervates dissolve. c) Schematic representation of the microfluidic chip that is used for microfluidic droplet formation and trapping of these droplets in dropspot chambers. EDC and precursor solutions are supplied from two different channels and are mixed right after the formation of the microfluidic droplets. Upon stopping the flow, the microfluidic droplets are trapped in the dropspot chambers. In these chambers, the microfluidic droplets are imaged via confocal time‐lapse imaging by excitation of sulforhodamine B for coacervate‐based droplets with pSS or Cy3‐A15 for coacervate‐based droplets with pU at 552 nm. The pseudocolor‐coded confocal image represents a maximum z‐projection of a z‐stack throughout one microfluidic droplet. The grey value scale from 0 to 255 is given next to the image. The grey line represents the periphery of the microfluidic droplet. The scale bar represents 20 μm.

To better capture this dynamic behavior by microscopy, we introduced a droplet‐generating microfluidic platform suitable for fuel‐driven self‐assembling systems (Figure 1c). The precursor and EDC solutions are injected via two different inlets in the design. Due to the laminar flow in the microfluidic device, no significant mixing happens before the encapsulation into the microfluidic droplets.^[22]^ Surfactant‐stabilized microfluidic water‐in‐perfluorinated oil droplets of equal size are produced at a T‐junction. The two fuel and precursor phases are homogenized directly after their encapsulation in the microfluidic droplet through convective mixing, which is considered the starting time of the fuel‐driven reaction cycle. In the following seconds, the microfluidic droplets pass through an array of so‐called dropspot chambers, which hold the microfluidic droplets in place once the flow of all inlets is stopped.^[23]^ To control the flow rates, we use a pressure controller instead of syringe pumps to ensure an almost immediate flow stop. Once the microfluidic droplets are entrapped in one of the dropspot chambers, they are imaged via confocal microscopy in an XYZ time series. Due to the minimal microfluidic droplet volume of 33 pl, it is possible to image the entire reaction volume via z‐stack imaging with a time resolution as short as 5 seconds per z‐stack (time‐resolution is limited by the image acquisition time of the microscope).

In other words, we can track the emergence, evolution, and decay of each coacervate‐based droplet in the microfluidic droplet at an interval down to 5 seconds. We first analyzed whether the droplets formed in bulk and microfluidic droplets behaved similarly. To create the pU‐based droplets in the microfluidic device, we combined the peptide stream with the fuel stream in a 1 : 1 ratio such that a 33±4 pl microfluidic droplet contained 23 mM peptide, 4.1 mM pU, and 25 mM fuel to form coacervate‐based droplets and 0.1 μM Cy3‐tagged A15 hybridized to pU to visualize them by confocal microscopy (Figure S1a–d, Figure S2a–d). The microfluidic droplet was captured less than 5 seconds after its creation and analyzed by confocal microscopy through sequential Z‐stack imaging (17.4 seconds per stack). Similar to experiments in bulk, in the first minutes, the droplets grew predominantly through fusion (Figure 2a, Movie S1, Figure S3b). Around 10 minutes into the cycle, the droplets started to form vacuoles, after which the droplets decayed and divided into smaller droplets (Figure S3c and d). Excitingly, because of the rapid mixing and imaging in microfluidics, we could, for the first time, see the nucleation of the droplets (Figure 2a, Figure S3a). We counted the number and measured the volume of each coacervate‐based droplet throughout the reaction cycle. The average droplet volume increased steadily in the first minutes due to the fusion‐induced growth (Figures 2b and c). After 10 minutes, it suddenly collapses due to droplet decay. Notably, the evolution of the average droplet volume and the total droplet volume was similar in microfluidic confinement and in bulk‐generated droplets.

**Figure 2:**
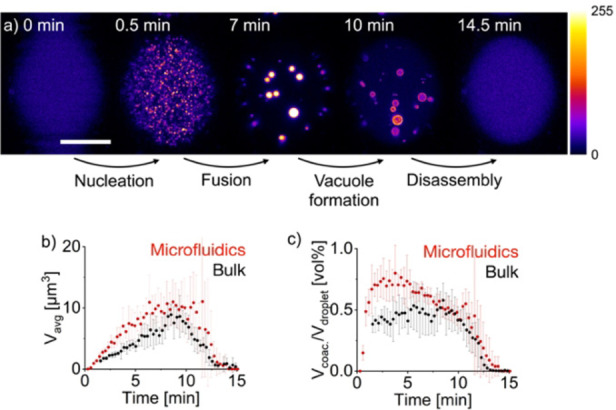
Comparison of the behavior of coacervate‐based droplets in the bulk and the microfluidic setup. Conditions are 23 mM Ac‐F(RG)_3_D‐OH, 4.1 mM pU, 0.1 μM Cy3‐A15 and 200 mM MES at pH 5.3 with 25 mM EDC. a) Representative images over one cycle of coacervate assembly and disassembly in a 33 pl water droplet entrapped in a microfluidic dropspot chamber. Images are recorded by excitation of Cy3‐A15 at 552 nm. The pseudocolor‐coded confocal image represents a maximum z‐projection of a z‐stack throughout one microfluidic droplet. The grey value scale from 0 to 255 is given next to the image. The scale bar represents 20 μm. b, c) Comparison of the average and the total volume of pU droplets between the microfluidic and the bulk setup. The total volume is given as volume percent, defined as the total volume of coacervate‐based droplets divided by the volume of the microfluidic droplet or the imaged volume for the bulk setup. Error bars represent the standard deviation of 5 independent experiments.

We only observed significantly dissimilar behavior if we used coacervate‐based droplets with pSS as the polyanion and if they reached diameters greater than a few micrometers. For example, when 10 mM peptide, 5 mM pSS, and 10 mM EDC were used, the diameter was relatively large, and we found that the droplet size of the coacervate‐based droplets grew faster in the confinement of microfluidic droplets than in bulk. (Figure S4a and b). We also observed that in these cases, almost all of the coacervate‐based droplets were lying at the bottom of the microfluidic droplet (Figure S4c and d). These observations can be explained by gravitationally induced fusion which has been reported previously in confined volumes,^[16]^ i.e., due to the small volume of their container, droplets settle and fuse at the increased local concentration at the bottom of their microfluidic container. This effect is less pronounced for pU coacervates, most likely, because pU coacervates are less dense than pSS coacervates (Figure S5a and b).

Due to the fast mixing and imaging possible, we observed, for the first time, the nucleation and growth of the coacervate‐based droplets (Figure 3a, Supporting Information Movie 2). The fluorescence was homogeneously distributed directly after the microfluidic droplet was created and captured in the device, but it coarsened with time. Within tens of seconds, droplets distinguishable from background fluorescence were detected. We define that time, i.e., when droplets with a diameter larger than 400 nm were detected, as the nucleation time. This definition is likely an overestimation of the actual nucleation due to the limited ability of light‐based microscopy to detect smaller particles. We analyzed the influence of the fuel, the peptide precursor, and the pSS concentration on the nucleation time. We observed that more coacervate‐based droplets were formed for higher EDC concentrations at lower nucleation times (Figure 3b and c, Figure S6g), i.e., the nucleation time decreases from 0.67±0.08 min for 10 mM EDC to 0.23±0.02 min for 30 mM EDC. We explain the observation because the anhydride concentration rises more rapidly and thus crosses the critical concentration for binodal or even spinodal decomposition.^[24]^ To verify this explanation, we used a previously written kinetic model. Briefly, the kinetic model calculates the concentration of all reagents of the reaction cycle for every second in the experiments through a set of differential equations. The model assumes a homogeneous solution, i.e., it does not consider the droplet material's phase separation. We can extract the kinetic model's kinetics of activation and deactivation, i.e., the activation constant *k*_1_ and the deactivation constant *k*_4_ (Supporting Information). Indeed, when we correlate the nucleation times with the anhydride concentrations predicted by the kinetic model, we find that nucleation occurred at an anhydride concentration of 0.61 mM, independent of the amount of fuel added (Figure 3d). Using this concentration, we can predict the dependence of the nucleation time on the fuel concentration with the kinetic model (Figure 3c), giving us a minimum concentration of 4.7 mM EDC needed to induce coacervation. Additionally, we found that with increasing precursor concentration, the nucleation time decreases (Figure S6a). The kinetics of the reaction cycle can also explain this observation since the activation reaction is a second‐order reaction and therefore scales both with the fuel and the precursor concentration. However, suppose we correlate the nucleation time again to the predicted anhydride concentrations. In that case, we observe that the anhydride concentration needed for nucleation decreases with increasing precursor concentrations (Figure S6b and h). We explain this because the precursor already has a particular affinity to the polyanion. Therefore, an increased precursor concentration reduces the amount of anhydride needed to induce coacervation. In contrast, increasing the pSS concentration increased the nucleation time because the amount of anhydride needed to induce coacervation scales with the pSS concentration (Figure S6d, e, and i).

**Figure 3:**
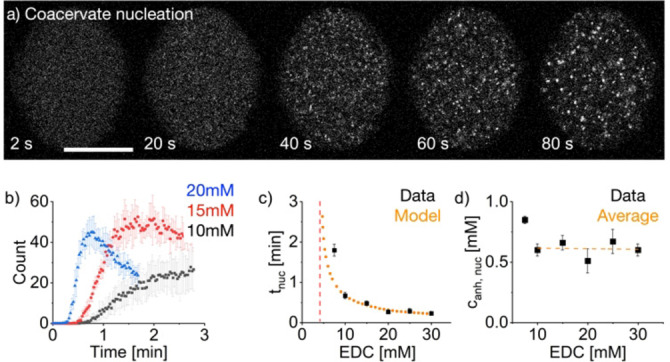
Analysis of coacervate‐based droplet formation. All experiments are performed with conditions of 8 mM Ac‐F(RG)_3_D‐OH, 5 mM pSS, 0.1 μM sulforhodamine B, and 200 mM MES at pH 5.3 with varying fuel concentrations. a) Representative images of coacervates’ initial formation and growth when 20 mM of EDC is added. Images are recorded by excitation of sulforhodamine B at 552 nm. The images show one z‐plane in the middle of a microfluidic droplet. The scale bar represents 20 μm. b) Number of coacervate‐based droplets depending on the amount of EDC added. Coacervate‐based droplets are counted in one z‐plane in the middle of a microfluidic droplet over the first 3 min after the start of the reaction cycle. Error bars represent the standard deviation of at least 9 measurements from 3 independent experiments. c) The time the first coacervate‐based droplet could be detected (*t*_nuc_) is shown as a function of the amount of EDC added. Error bars represent the standard deviation of at least 9 measurements from 3 independent experiments. The orange dotted line represents the nucleation times calculated by the kinetic model depending on the EDC concentration. The red line represents the EDC concentration below which no fuel‐driven nucleation of coacervate‐based droplets is possible according to the kinetic model. d) Anhydride concentration for different EDC concentrations at the time of nucleation. The kinetic model calculates anhydride concentrations. The orange dotted line represents the average anhydride concentration needed to nucleate coacervate‐based droplets. Error bars are calculated from the standard deviation of the nucleation times.

There are different methods to determine the total volume of the separated phase, like centrifugation^[13a]^ or confocal microscopy.^[25]^ Centrifugation requires sample volumes of several 100 μL for accurate determination. In contrast, confocal microscopy suffers from an inhomogeneous distribution of the coacervate‐based droplets, especially in the z‐direction throughout the sample. Through encapsulation into microfluidic droplets, the inhomogeneous distribution in the z‐direction can be overcome, but time‐resolved measurements remain challenging.[^8^, ^16^] With the microfluidic setup presented, it is possible to determine the total volume of the phase‐separated droplets, as a function of time, from their nucleation until their dissolution. First, we compared the total volumes measured by centrifugation to those measured in the microfluidic setup. To avoid errors due to the time‐dependence of the total volume in fuel‐driven LLPS systems, we used a static LLPS‐system. Specifically, we used Ac‐F(RG)_3_N‐NH_2_, i.e., a mimic of our active product that is permanently 3+ because its carboxylates are amidated. We confirmed that the total volumes measured with our microfluidic setup matched the total volumes measured through established methods (Figure S7a and b).

Next, we used the active droplets and tested how the fuel, the peptide precursor, and the pSS concentration influenced the maximum total volume of the coacervate‐based phase. The amount of fuel added determined the total volume of coacervate‐based droplets (Figure 4a). However, quantitative analysis showed that the total volume only increased until an EDC concentration of about 20–25 mM, after which it, surprisingly, leveled off (Figure 4b and c, Figure S8a). The complete conversion of the precursor cannot explain the decline in the anhydride (Figure S6g), and centrifugation of the non‐fuel‐driven LLPS system with different pseudo‐anhydride to precursor ratios confirmed that upon reaching a specific precursor conversion, the total volume of coacervate‐based droplets is not increasing further (Figure S8d). Moreover, we observed that the time coacervate‐based droplets remained non‐spherical after fusion increased with higher EDC concentrations (Figure 4e, Figure S9a and b). Both observations can be explained by increased viscosity of the droplet phase with increasing fuel, i.e., a denser phase would imply a smaller volume and slower droplet fusion. FRAP experiments on the diffusivity of NBD‐labeled precursor confirmed that the diffusivity inside the coacervate‐based droplets decreased with increasing EDC (Figure 4d, Figure S10a–d). To verify that the decrease in diffusivity results from the peptide product and is not induced directly by the EDC itself, we performed similar FRAP experiments on the non‐dynamic coacervate‐based droplets with the static Ac‐F(RG)_3_N‐NH_2_ as peptide (Figure S8c). We observe the same behavior for the dye sulforhodamine B (Figure S8b). Those experiments confirmed that diffusivity decreases by increasing the peptide to precursor ratio until no more liquid droplets are formed. Instead, a more solid‐like precipitate is observed. We hypothesize that from around 25 mM EDC, no further polyanion is recruited into the coacervate‐based droplets upon further increasing the fuel concentration. Further increasing the fuel, and thus the amount of anhydride leads to replacing precursor with anhydride molecules in the coacervate‐based droplets. The higher affinity of anhydride for pSS leads to a denser packing of the coacervate‐based droplets and hence a stagnation in the total volume increase. For verification, we conducted fluorescence partitioning experiments with non‐dynamic droplets using Ac‐F(RG)_3_N‐NH_2_ as a model peptide for the anhydride. For non‐dynamic coacervate‐based droplets, we observed that the pSS recruitment into the droplets only increased up to 2 mM of the model anhydride and leveled off afterward (Figure S11a).

**Figure 4:**
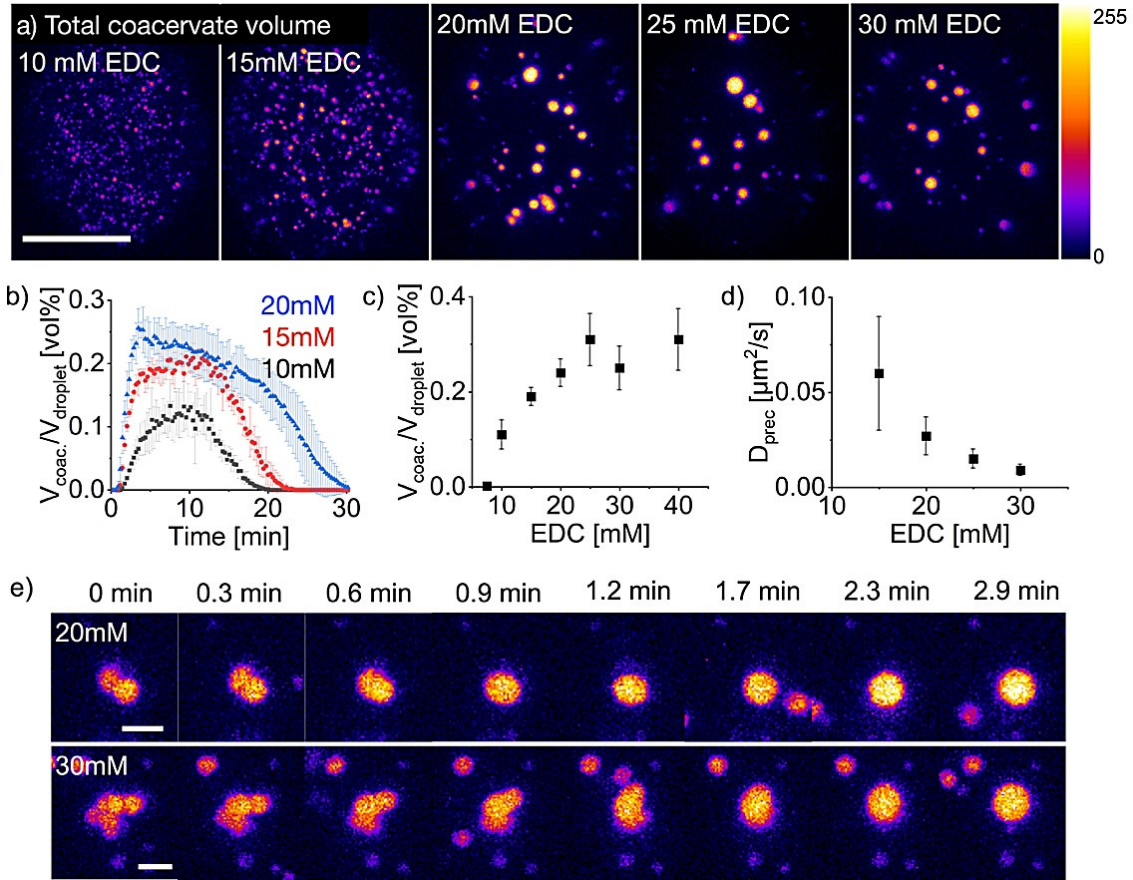
Analysis of the total volume of coacervate‐based droplets and their viscosity. All conditions are 8 mM Ac‐F(RG)_3_D‐OH, 5 mM pSS, and 200 mM MES at pH 5.3 with varying fuel concentrations. a) Images of the coacervation cycle showing the maximum amount of coacervate volume at different EDC concentrations. Images are recorded by excitation of sulforhodamine B at 552 nm. The pseudocolor‐coded confocal images represent a maximum z‐projection of a z‐stack throughout one microfluidic droplet. The grey value scale is given next to the images. The scale bar represents 20 μm. b) Analysis of the total volume of coacervate‐based droplets over the entire reaction cycle. The total volume is given as volume percent, defined as the total volume of coacervate‐based droplets divided by the total volume of the microfluidic droplet. Error bars represent the standard deviation of 3 experiments. c) Maximum total volume of coacervate‐based droplets as a function of the EDC concentration. Error bars represent the standard deviation of 3 experiments. d) The diffusivity of NBD‐labeled product inside of coacervate‐based droplets. Error bars represent the standard deviation of 9 experiments. e) Time series of droplet fusion. Fusion is drastically slower at increased EDC concentrations. The depicted time represents the time of the fusion event and not the actual time in the reaction cycle. Images are recorded by excitation of sulforhodamine B at 552 nm. The scale bar represents 2 μm.

In contrast, the precursor recruited into the coacervate‐based droplets decreased with increasing model anhydride concentrations (Figure S11b). We observed similar total peptide amounts recruiting into the coacervate‐based droplets for the fuel‐driven system for 20 mM and 30 mM EDC (Figure S11c). These experiments conclude that the anhydride to precursor ratio in the droplets increased. Thus, more fuel leads to more droplet material, but it also leads to a denser packing of the droplet material.

A significant advantage of our experimental setup is that each coacervate‐based droplet can be followed throughout its lifetime. In perspective, droplets prepared in larger containers are impossible to track due to Brownian motion in and out of the imaging area. Thus, we tracked the emergence, motion, fusion, and decay of an entire population of coacervate‐based droplets for the first time. We used automated particle‐tracking^[26]^ and manually optimized the trajectory linking and tracing. In the first 1.5 min, despite the small volume of the microfluidic droplet, it was impossible to reliably track individual coacervate‐based droplets because of their small size and fast movement. From there on, we could identify 39 coacervate‐based droplets, and we tracked their motion, fusion events, and decay pathways (Figure 5a and b, Supporting Information Movie 3). From the collective data, we observed several new behaviors. First, we found that coacervate‐based droplets fused more frequently than we expected, i.e., all 39 droplets fused more than once until only three droplets remained at 12.6 minutes. We color‐coded these three droplets and the droplets they originated from before fusion events cyan, orange and green. The droplets fused 19 (orange), 13 (cyan), and 3 (green) times, respectively. We found that coacervate‐based droplets did not move far between fusion events. In other words, the last three droplets were roughly in the center of all of their original droplets. This confirms there is no convective flow inside the microfluidic droplet, and the coacervates move exclusively by Brownian motion.

**Figure 5:**
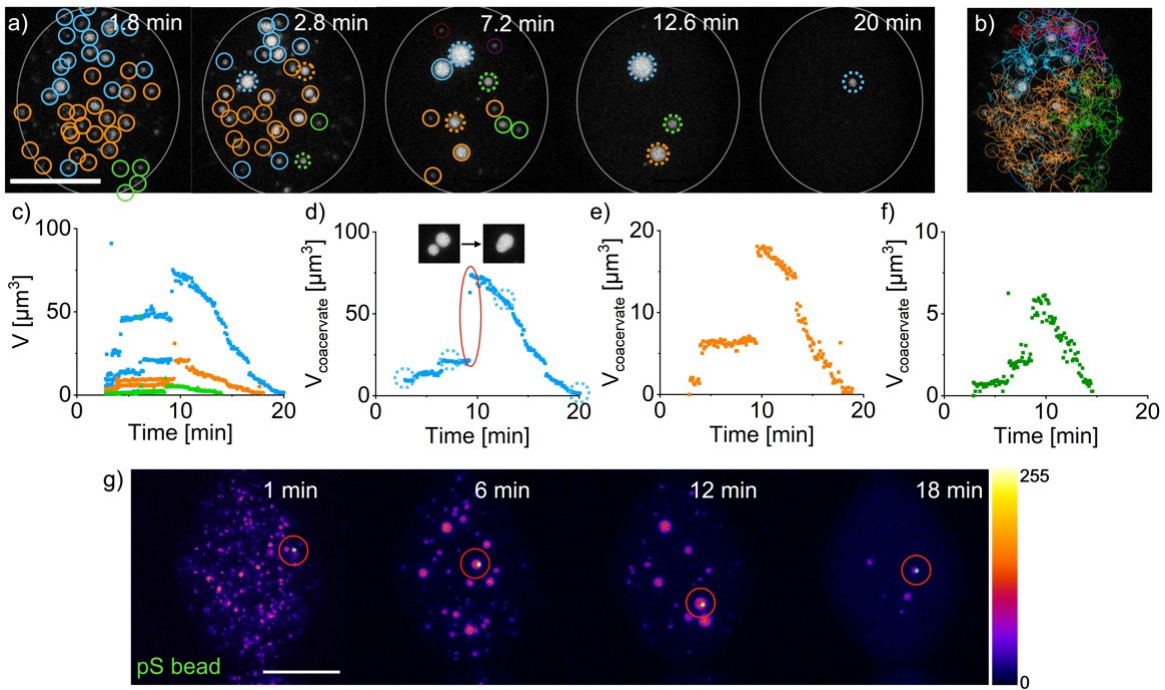
Tracking of coacervate‐based droplets in a microfluidic droplet. a) A z‐projection image time series. All droplets that fuse are assigned to the same population and marked with the same color. A solid grey circle marks the periphery of the microfluidic droplet. The dotted circles mark the coacervate‐based droplet that is tracked under (d)–(f). b) The combined pathways of every droplet of the different populations. c) Volumes of every coacervate‐based droplet of the different populations detected in the microfluidic droplet. d)–f) The volume of one coacervate‐based droplet the population picked at 2.8 min and followed until its dissolution. A fusion event is highlighted. g) The same experiment as above, but with a blue‐fluorescent pS bead. The bead incorporates into a coacervate‐based droplet and remains incorporated until its dissolution. The grey value scale is shown next to the images. All scale bars represent 20 μm. All conditions are 10 mM Ac‐F(RG)_3_D‐OH, 5 mM pSS and 0.1 μM sulforhodamine B in 200 mM MES at pH 5.3.

The droplets’ tracking data allowed us to follow the “life” of every individual droplet, which offered insights into the behaviors of a droplet on an individual level. For example, the individual volume of every coacervate is tracked (Figure 5c). In that plot, we color‐coded the data points based on the color‐coding of the last three droplets (cyan, orange, and green). To make the plot comprehensible, we only show 12 droplets. When a track remains horizontal, the droplet neither grows nor shrinks, while a sudden increase in the track represents a fusion event. From these tracks, we can conclude that between 2 to 10 minutes, the droplet size is relatively stable, i.e., they do not grow or shrink.

The primary mechanism of growth in this timeframe is fusion. Coalescence has been reported as the primary mechanism for droplet growth.^[16]^ It is in line with recent findings that coacervate‐based droplets do not undergo major Ostwald ripening, or ripening can even be suppressed.^[27]^ Then, after 10 minutes, all three remaining droplets start to shrink following an exponential decay until the droplet size falls below the detection limit of the tracking software. In the decay profile of the droplets, we found another new behavior. The rate of the decay of the droplets scales with the droplet volume. The greater the volume, the faster the droplets decay. This first‐order decay seems to suggest that the pseudo‐first‐order of the volume loss per unit of time, i.e., the slope of these lines, is the same for each of the three remaining droplets (Figure S12a and b), which indicates that the hydrolysis of the anhydride is the rate‐determining step for the droplet decay and not the disassembly of the precursor molecules. Additionally, the exponential volume decay can be captured well using only the kinetic model's anhydride hydrolysis constant (*k*_4_). The calculated volume decay slightly overestimates the measured volume decay, most likely due to a residual activation that is still happening in the system (Figure S12b). The correlation between the anhydride hydrolysis and the loss of droplet material was strong even at high EDC concentrations, where droplets become more viscous (Figure S13a). We explain this observation because the reduced diffusivity at high EDC concentrations is not constant throughout the reaction cycle but tends to increase again at the end of the cycle. Therefore, coacervate‐based droplets get more liquid towards the end of the reaction cycle when the amount of activated species is reduced, and no diffusion limitation is observed for the dissolution of the coacervate‐based droplets. The decrease in the viscosity and, therefore, the increase in the diffusivity throughout the cycle can be observed by a reduction in the fusion time from 1.5 min at the start of the reaction cycle to less than 0.3 min right before the dissolution of the coacervate‐based droplets occurs (Figure S13b). The close resemblance of volume decay and anhydride decay emphasizes that our coacervate‐based droplets are dynamic. The identical shrinkage rate constant also leads to a longer lifetime the bigger the coacervate‐based droplets are, as they can maintain a sufficient size for a more extended period.

We can also analyze the average likelihood of fusion for coacervate‐based droplets depending on the EDC concentration (Figures S14a and b). Here, we observe that the fusion rate scales with the EDC concentration in the first minutes. We explain this trend by faster growth in the average volume of the coacervate‐based droplets and the resulting higher likelihood of fusion. For fuel concentrations above 20 mM EDC the fusion rate declines significantly after 5 minutes. In comparison, experiments with fuel concentrations below 20 mM EDC show a more homogeneous fusion rate throughout the reaction cycle. For high EDC concentrations, the rapid decrease in the number of coacervate‐based droplets and their decreased mobility due to their bigger average size leads to a decreased likelihood of droplet fusion.

When we followed the life of a single droplet, we found that it underwent three major fusion events between 3 and 12 minutes (Figure 5d). Each of the three fusion events was characterized by a jump in its volume which further corroborates the growth‐through‐fusion hypothesis. This fusion behavior was observed for most droplets (Figures 5e and f). This setup makes it possible to analyze the collective behavior of coacervate‐based droplets and each droplet‘s individual “life”. Furthermore, we believe that tracking individual coacervates opens the possibility of future selection or evolution‐based studies. This could, for example, be achieved by introducing one enzyme‐coated micrometer‐sized particle per reaction chamber that is incorporated into the coacervate‐based droplets. To demonstrate this feature, we introduced a single polystyrene bead (pS bead) into the microfluidic droplets (Figure 5g). A coacervate‐based droplet takes up this unfunctionalized pS bead and remains in the droplet until dissolution. The unfunctionalized pS beads do not affect the behavior of the coacervate‐based droplet. If the surface of this bead were catalytically active, its activity would only alter the behavior of this single droplet, giving it a potential advantage or disadvantage over others.

We successfully imaged the entire life‐cycle of fuel‐driven active droplets enabled by a microfluidic system for droplet formation and analysis. With the method, we found four new behaviors in our active droplets. The droplets nucleate rapidly, but the kinetics of the reaction cycle can tune the nucleation time, i.e., the nucleation time directly follows the rate of anhydride formation. The droplets grow because of fusion, they fuse frequently, and their frequency of fusion at the beginning of the reaction cycle scales with the fuel concentration. However, the droplet's density increases with an increasing amount of fuel added, making the fusion process slower. The droplets decay at roughly the same timepoint, the shrinkage rate constant is independent of their volume and is equal to the hydrolysis rate constant of the activated species.

The advantages of studying our droplets in microfluidics combined with a dropspot chamber can be used with other active systems. We anticipate that the method will be applied to study other chemically fueled systems in more detail. We also anticipate that this setup will be handy for evolution‐based experiments where droplets will compete for scarce resources and the future observation of self‐dividing or self‐reproducing droplets systems.

## Conflict of interest

The authors declare no conflict of interest.

## Supporting information

As a service to our authors and readers, this journal provides supporting information supplied by the authors. Such materials are peer reviewed and may be re‐organized for online delivery, but are not copy‐edited or typeset. Technical support issues arising from supporting information (other than missing files) should be addressed to the authors.

Supporting Information

Supporting Information

Supporting Information

Supporting Information

## Data Availability

The data that support the findings of this study are available from the corresponding author upon request.
