# Supplementary material for: Evolution and Single‐Droplet Analysis of Fuel‐Driven Compartments by Droplet‐Based Microfluidics
Source: Angew Chem Int Ed Engl. 2022 Jun 24;61(32):e202203928. doi: 10.1002/anie.202203928 (PMC9400878; doi:10.1002/anie.202203928)
Supplement: Supplementary file 1 — Supporting Information [file ANIE-61-0-s004.pdf]

Angewandte Chemie
Eine Zeitschrift der Gesellschaft Deutscher Chemiker

Supporting Information

Evolution and Single-Droplet Analysis of Fuel-Driven Compartments by Droplet-Based Microfluidics

A. M. Bergmann, C. Donau, F. Späth, K. Jahnke, K. Göpfrich, J. Boekhoven**

Experimental Section

Materials. All solvents were purchased in analytical grade from Sigma Aldrich and used without further purification. N,N-Dimethyl formamide (DMF) was purchased from Sigma-Aldrich in peptide synthesis grade. Fmoc-R(Pbf)-OH, Fmoc-D(OtBu)-OH, Ac- F-OH, Fmoc-G-OH and Fmoc-N(Trt)-OH), N,N'-Diisopropylcarbodiimide (DIC), Ethyl cyano(hydroxyimino)acetate (Oxyma, Nova-biochem©), Wang resins (100- 200 mesh, 0.4-0.8 mmol/g), Rink Amide resins (100- 200 mesh, 0.4-0.8 mmol/g), 4-(Dimethylamino)-pyridine (DMAP), Trifluoroacetic acid (TFA, 99%), Piperidine (99%), Triisopropylsilane (TIPS), N,N-Diisopropylethylamine (DIPEA), 4- Chloro-7-nitrobenzofurazan (NBD-Cl, 98%), styrene sulfonate sodium salt 2-(Dodecylthiocarbonothioylthio)-2-methylpropionic acid (98%), 4,4'-Azobis(4-cyanovaleric acid (ACPA, ≥98%), tris(2-carboxyethyl) phosphine (TCEP), sodium borohydride (NaBH4), Polystyrene sulfonate (pSS, 75kDa, 18wt% in water), polyuridylic acid potassium salt (pU), 1-ethyl-3-(3-dimethylaminopropyl)carbodiimide (EDC), sulforhodamine 101, 4-morpholineethanesulfonic acid (MES) buffer and Sylgard 184 silicon elastomere were all purchased from Sigma-Aldrich and used without any further purification unless indicated otherwise. The peptide Ac-F(RG)3D-OH was purchased from CASLO Aps. Nuclease-free water was freshly prepared by filtration (DURAN) of MQ-water. Cy3-A15 was purchased from biomers.net GmbH. 2w% 008-Fluorosurfactant in HFE7500 was purchased from RAN Biotechnologies. Sulfo-Cyanine5-maleimide(1-(6-((2-(2,5-dioxo-2,5-dihydro-1H-pyrrol-1-yl)ethyl)amino)-6-oxohexyl)-3,3-di-methyl-2-((1E,3E)-5-((E)-1,3,3-trimethyl-5-sulfonatoindolin-2-ylidene)penta-1,3-dien-1-yl)-3H-indol-1-ium-5-sulfonate) was purchased from Lumiprobe.

Peptide synthesis and purification. The peptides Ac-F(RG)$_3$N-NH$_2$ and NBD-G(RG)$_3$D-OH were synthesized via solid-phase synthesis on a CEM Liberty microwave-assisted peptide synthesizer, purified by reversed-phase HPLC and characterized by electrospray ionization mass spectrometry (ESI-MS) as well as analytical HPLC as previously described.[1]

Synthesis of Cy5-pSS. Poly(styrene sulfonate) homopolymer was synthesized via RAFT polymerization. For this purpose, styrene sulfonate sodium salt was polymerized in MQ-water at 75°C with 0.01 eq. 2-(Dodecylthiocarbonothioylthio)-2-methylpropionic acid (98%) and 0.002 eq. of 4,4'-Azobis(4-cyanovaleric acid (ACPA, ≥98%) for 5 hours. The solution was purged before with Argon for 20 minutes to remove air. The crude solution was purified by dialysis against MQ-water in 12-14 kDa MWCO dialysis tubings (Spectra/Por© 4, 29 mm diameter) for two days. The product was dried by lyophilization. The CTA-terminated poly(styrene sulfonate) (440 mg) was solubilized in 15 mL MQ-water, 15 mL of a freshly prepared 1M NaBH4 solution were added dropwise and the mixture was then

stirred for 3.5 hours at room temperature. The crude solution was dialyzed against MQ-water for 2 days in 12-14 kDa MWCO dialysis tubings (Spectra/Por© 4, 29 mm diameter) and lyophilized. The resulting product was then reacted with 150 eq. of tris(2-carboxyethyl) phosphine (TCEP) at room temperature in DMF for 24 hours. 20 eq. of Sulfo-Cyanine5-maleimide(1-(6-((2-(2,5-dioxo-2,5-dihydro-1H-pyrrol-1-yl)ethyl)amino)-6-oxohexyl)-3,3-di-methyl-2-((1E,3E)-5-((E)-1,3,3-trimethyl-5-sulfonatoindolin-2-ylidene)penta-1,3-dien-1-yl)-3H-indol-1-ium-5-sulfonate) were added to an aliquot of this solution together with a catalytic amount of ethylenediamine and stirred at 50 °C for 24 hours. The crude product was purified by dialysis in a 2000 MWCO Slide-A-Lyzer Dialysis Cassette© (ThermoFisher Scientific) against MQ-water for 2 days and finally lyophilized.

Standard sample preparation. Stock solutions of Ac-F(RG)$_3$-D-OH (300 mM), pU (41 mM, according to monomer units), pSS (41 mM, according to monomer units) and MES (650 mM) were prepared by dissolving the respective amount of each component in MQ water and adjusting the pH to 5.3 by addition of a 5 M sodium hydroxide solution. Stock solutions for NBD-G(RG)$_3$D-OH, Ac-F(RG)$_3$N-NH$_2$, sulforhodamine B, Cy3-A15 and Cy5-pSS were also prepared in MQ water but without pH adjustment. All stocks were filtrated with a syringe filter (PTFE, 0.2µm pore size). Samples with the desired concentrations of each component were prepared from these stock solutions. For the preparation of non-fuel-driven coacervate-based droplets, the added concentration of the pseudo anhydride Ac-F(RG)$_3$N-NH$_2$ is subtracted from the precursor Ac-F(RG)$_3$-D-OH to keep the total peptide concentration constant. Stock solutions for EDC were prepared freshly before each experiment by dissolving the respective amount in MQ water.

Microfluidic chip production. Microfluidic PDMS (Polydimethylsiloxane, Sylgard 184, Dow Corning)-based devices were designed with QCAD-pro (RibbonSoft GmbH) and fabricated using photo- and soft-lithography[2] as previously described.[3]

Microfluidic droplet formation. Surfactant-stabilized water in oil droplets was produced using 2% 008-FluoroSurfactant in 3M Novec7500 as the oil phase, EDC in MQ water as one of the water phases and Ac-F(RG)3D-OH, PSS/pU and sulforhodamine B in MES buffer at pH 5.3 as the second water phase. Both water phases contained the doubled concentrations of the respective components and were mixed in a 1 to 1 ratio to yield the desired concentrations inside the microfluidic droplets. Both aqueous phases and the oil phase were injected into the microfluidic PDMS-based device through polytetrafluoroethylene (PTFE) tubes (0.4-0.9 mm, Bola, Germany) using a flow control system (ELVEFLOW Pressure Controller OB1 MK3). Typically, pressures of 610 mbar for the water phases and 810 mbar for the oil phase were used to produce stable water in oil droplets with a diameter of 40 µm.

Confocal fluorescence microscopy. To analyze the coacervate in a microfluidic droplet a lightning SP8 confocal microscope (Leica) with a 63x water immersion objective was used. Coacervates were stained with the fluorophore sulforhodamine B which was excited at 552 nm and detected from 565-700 nm with a HyD detector. The pinhole was set to 1 Airy unit. To analyze the evolution of coacervates in the entire microfluidic droplet, time series imaging of a single microfluidic droplet was acquired in z-stacks with 2 µm between z-planes (20 z-planes in total). All experiments that imaged the entire microfluidic droplet were acquired with a resolution of 600 x 600 pixels at 3.3x zoom and 400x scan speed (bidirectional scan) which results in a time resolution of 17.4 s/stack. To get a better time resolution for the tracking of the coacervates inside of the microfluidic droplet, the scan speed was increased to 1800x which results in a time resolution of 5.4 s/stack. To analyze the nucleation of coacervates, one z-plane of four microfluidic droplets was imaged simultaneously over time. All experiments that imaged the nucleation of coacervates were acquired with a resolution of 1334 x 1334 pixels at 1.43x zoom and 400x scan speed (bidirectional scan) which results in a time resolution of 1.4 s/frame.

Fluorescence recovery after photobleaching. The diffusivity inside of coacervates was calculated from fluorescence recovery after photobleaching experiments. Measurements were performed in a microscopy well plate chamber (IBIDI, µ-slide Angiogenesis Glass Bottom). Coacervates were stained with sulforhodamine B or NBD-G(RG)$_3$D-OH, an NBD labeled fluorescent version of the precursor peptide Ac-F(RG)$_3$D-OH, which was excited at 488 nm and detected from 565-635 nm with a PMT detector. FRAP experiments were performed on coacervates that were close to the glass at the bottom of the imaging chamber to have coacervates of sufficient size and to minimize their movement during image acquisition. Images were acquired at a resolution of 135x135 pixels, 400x scan speed (bidirectional scan) and 15x zoom. A spot size of 1 µm in diameter was chosen for bleaching and is kept constant for all experiments. Recovery data were normalized through double normalization with the following equation:[4]

$$F(t) = \frac{(T_0 - B_o)(I_t - B_t)}{(T_t - B_t)(I_0 - B_0)}$$

Here *F(t)* represents the normalized fluorescence intensity which is calculated from the average intensity of 3 ROIs. I_t represents the average intensity of the bleached ROI, T_t represents the average intensity of an unbleached ROI within the bleached coacervate and B_t represents the average intensity of a ROI without any coacervates. The fluorescence recovery *F(t)* is then given by the equation[5]

$$F(t) = F_{\infty} \exp\left[-\frac{2}{1+\left(\frac{8tD}{a^2}\right)}\right]$$

with F_{∞} representing the fluorescence at full recovery, *a* representing the bleached area and *D* representing the translational diffusion coefficient of the fluorescent probe.[5]

Image analysis. The z-stack image-series obtained by confocal microscopy are converted into maximum z-projections using Fiji ImageJ. One threshold was determined with the "moments"[6] algorithm for each different set of imaging parameters to separate the fluorescence of the coacervate-based droplets from the background fluorescence. The "watershed" plugin is used to separate detected non-spherical particles resulting from overlapping fluorescence of two or more coacervate-based droplets. The preinstalled "analyze particles" package of Fiji ImageJ is used to determine the number and area of the coacervate-based droplets. The volume is calculated from the area of coacervate-based droplets under the assumption that they are perfectly spherical.

Tracking of coacervates. Tracking of coacervate-based droplets was done with the imageJ plugin TrackMate.[7] The parameters used for tracking of every coacervate were LoG detector with an estimated diameter of 3.2µm, a threshold of 0.09 and LAP Tracker with a frame to frame linking of 5 µm, gap closing of 5 µm and 2 frames, and segment merging of 15 µm. The parameters used for volume tracking were LoG detector with an estimated diameter of 4.5µm, a threshold of 0.1 and LAP Tracker with a frame to frame linking of 5 µm, gap closing of 5 µm and 2 frames, and segment merging of 5 µm. Subsequently, the tracks were inspected and corrected manually. The estimated blob diameter for volume tracking was increased to more accurately display the diameter of the bigger coacervate-based droplets while a lower estimated blob diameter gave better results for the path tracking of small coacervate-based droplets.

Centrifugation. For centrifugation samples of 200 µl were prepared as described previously. To improve the contrast between coacervate- and dilute-phase, 15 µM of sulforhodamine B was added to each sample. The samples were incubated for 15 min and then centrifuged for another 15 min at 13.5k rpm. The volumes of the coacervate phases were determined by comparison with samples of known sulforhodamine volumes.

Fluorescence spectroscopy. Fluorescence spectroscopy was performed on a Jasco spectrofluorimeter (Jasco FP-8300, SpectraManager software 2.13) with external temperature control (Jasco MCB-100). To determine the amount of NBD-labeled peptide in the dilute phase of fuel-driven coacervate-based droplets, 150 µl samples of 8 mM Ac-F$(RG)_3$D-OH, 5 mM pSS, and 1 µM NBD-G$(RG)_3$D-OH in 200 mM MES buffer at pH 5.3 were prepared. As a

control, samples of the same conditions without NBD-G$(RG)_3$D-OH were prepared. EDC is added to the samples and after 4 min they were centrifuged for 5 min at 20,412 g. 100 µl of the clear supernatant solution is carefully removed with a pipette. To account for the dependence of the fluorescence of dyes on their environment, 1 µM of NBD-G$(RG)_3$D-OH is added to the control samples after centrifugation. The samples were excited at 467 nm and the fluorescence intensity at 526 nm in comparison to the corresponding control sample gave the remaining amount of NBD-G$(RG)_3$D-OH in the dilute phase.
To determine the amount of NBD-labeled precursor and Cy5-labeled pSS in the dilute phase of non-fuel-driven coacervate-based droplets, 150 µl samples with 8 mM peptide as a combination of Ac-F$(RG)_3$D-OH and Ac-F$(RG)_3$D-NH_2, 5 mM pSS, and 1 µM NBD-G$(RG)_3$D-OH or 1 µM Cy5-pSS in 200 mM MES buffer at pH 5.3 were prepared. As a control, samples of the same conditions without the fluorophore were prepared. To induce coacervation, pSS is added at last to the samples and after 10 min they were centrifuged for 15 min at 20,412 g. 100 µl of the supernatant solution is carefully removed with a pipette and added to a centrifugal tube containing 1 µl of an aqueous solution of NaCl (4 M) to dissolve residual turbidity. Similar to the fuel-driven system, the respective fluorophore is added to the control samples after centrifugation. The samples with NBD-G$(RG)_3$D-OH were excited at 467 nm and the fluorescence intensity at 526 nm in comparison to the corresponding control sample gave the remaining amount of peptide in the dilute phase. The samples with Cy5-pSS were excited at 630 nm and the fluorescence intensity at 653 nm in comparison to the corresponding control samples gave the remaining amount of pSS in the dilute phase.

Kinetic model. We used a kinetic model written in MATLAB to predict the evolution of the anhydride concentration over time. The rate constants were determined by fitting the model to peptide anhydride, EDC, and precursor concentrations measured by analytical HPLC. More details can be found in our previous work.[1] The following rate constants were used

k0 = 7.50 · 10-5 s-1
k1 = 1.80 · 10-1 M-1 s-1
k2 = 5.40 · 10-1 s-1
k3 = 2.16 · 10-1 s-1
k4 = 0.85 · 10-2 s-1 [1a]

Supporting Figures

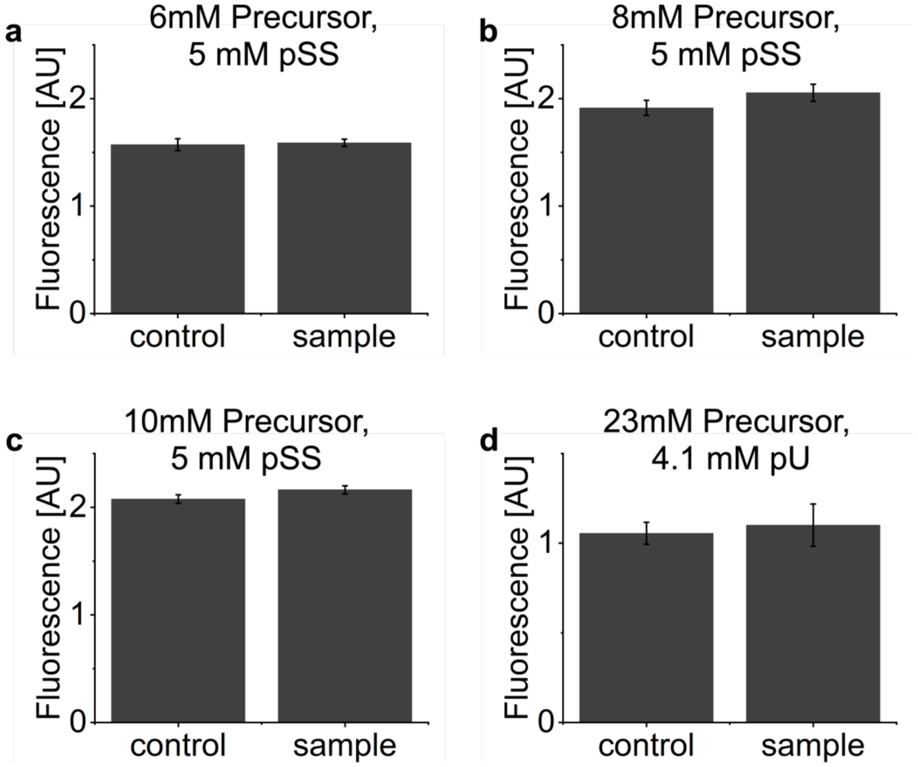

Supporting figure 1. Verification of droplet composition. a-d *Average fluorescence of 0.1 µM sulforhodamine B for pSS and 0.1 µM Cy3-A15 for pU measured inside of the microfluidic droplets. For the samples, precursor, pSS/pU, MES and sulforhodamine B/Cy3-A15 were prepared with double the concentration as indicated and were then mixed in the microfluidic device with water in a 1:1 ratio and encapsulated into microfluidic droplets. For the control, a sample with the indicated concentrations of precursor, pSS/pU, MES and sulforhodamine B/Cy3-A15 were encapsulated into microfluidic droplets without mixing with water. The fluorescence is compared to verify correct mixing ratios. Error bars represent the standard deviation from triplicate measurements.*

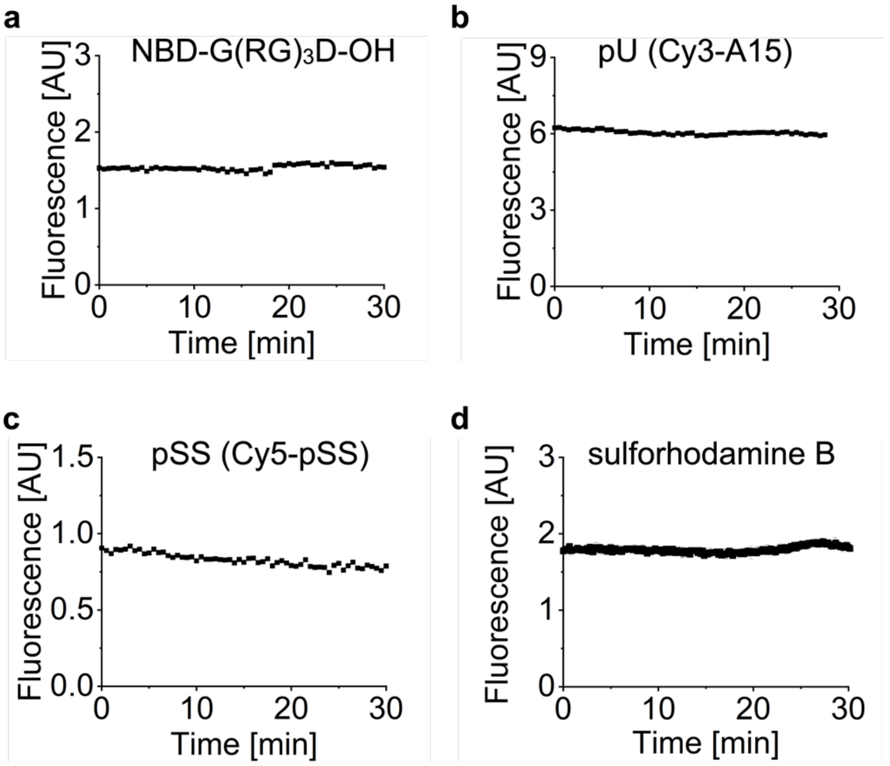

Supporting figure 2. Concentration stability of the fluorescent components of coacervate-based droplets. a *Fluorescence of 1 µM NBD-G(RG)$_3$D-OH was measured over time inside of a microfluidic droplet. Conditions are 10 mM Ac-F(RG)$_3$D-OH, 5 mM pSS, 200 mM MES at pH 5.3.* ***b*** *Fluorescence of 0.1 µM Cy3-A15 was measured over time inside of a microfluidic droplet. Conditions are 23 mM Ac-F(RG)$_3$D-OH, 4.1 mM pU, 200 mM MES at pH 5.3.* ***c*** *Fluorescence of 1 µM Cy5-pSS was measured over time inside of a microfluidic droplet. Conditions are 10 mM Ac-F(RG)$_3$D-OH, 5 mM pSS, 200 mM MES at pH 5.3.* ***d*** *Fluorescence of 0.1 µM sulforhodamine B was measured over time inside of a microfluidic droplet. Conditions are 10 mM Ac-F(RG)$_3$D-OH, 5 mM pSS, 200 mM MES at pH 5.3.*

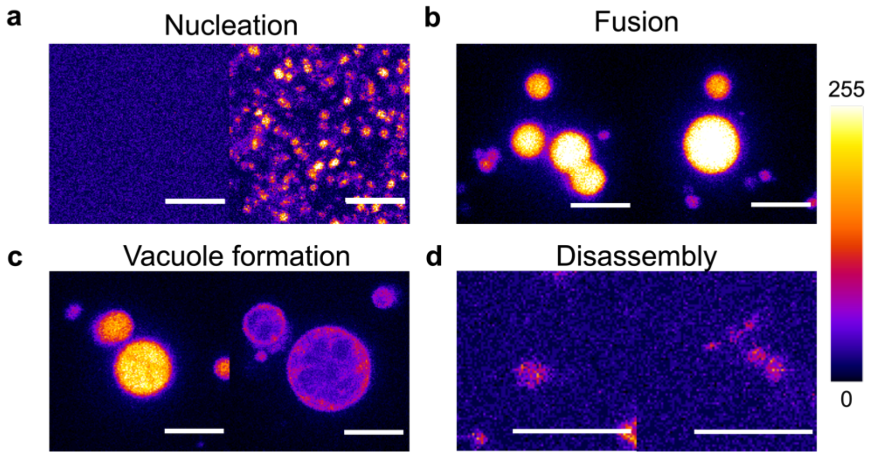

Supporting figure 3. Different behaviors of coacervate-based droplets. *Conditions are 23 mM Ac-F(RG)$_3$D-OH, 4.1 mM pU, 200 mM MES at pH 5.3 with 25 mM EDC. The scale bar represents 5 µm. The pseudocolor-coded confocal image represents a maximum z-projection of a z-stack throughout one microfluidic droplet. The grey value scale from 0 to 255 is given next to the images.* ***a*** *Formation of coacervate-based droplets after the addition of EDC.* ***b*** *Fusion of coacervate-based droplets with each other and the recovery of a spherical shape after fusion.* ***c*** *Coacervate-based droplets form vacuoles during their decay.* ***d*** *The dissolution of coacervate-based droplets leads to the formation of small daughter droplets.*

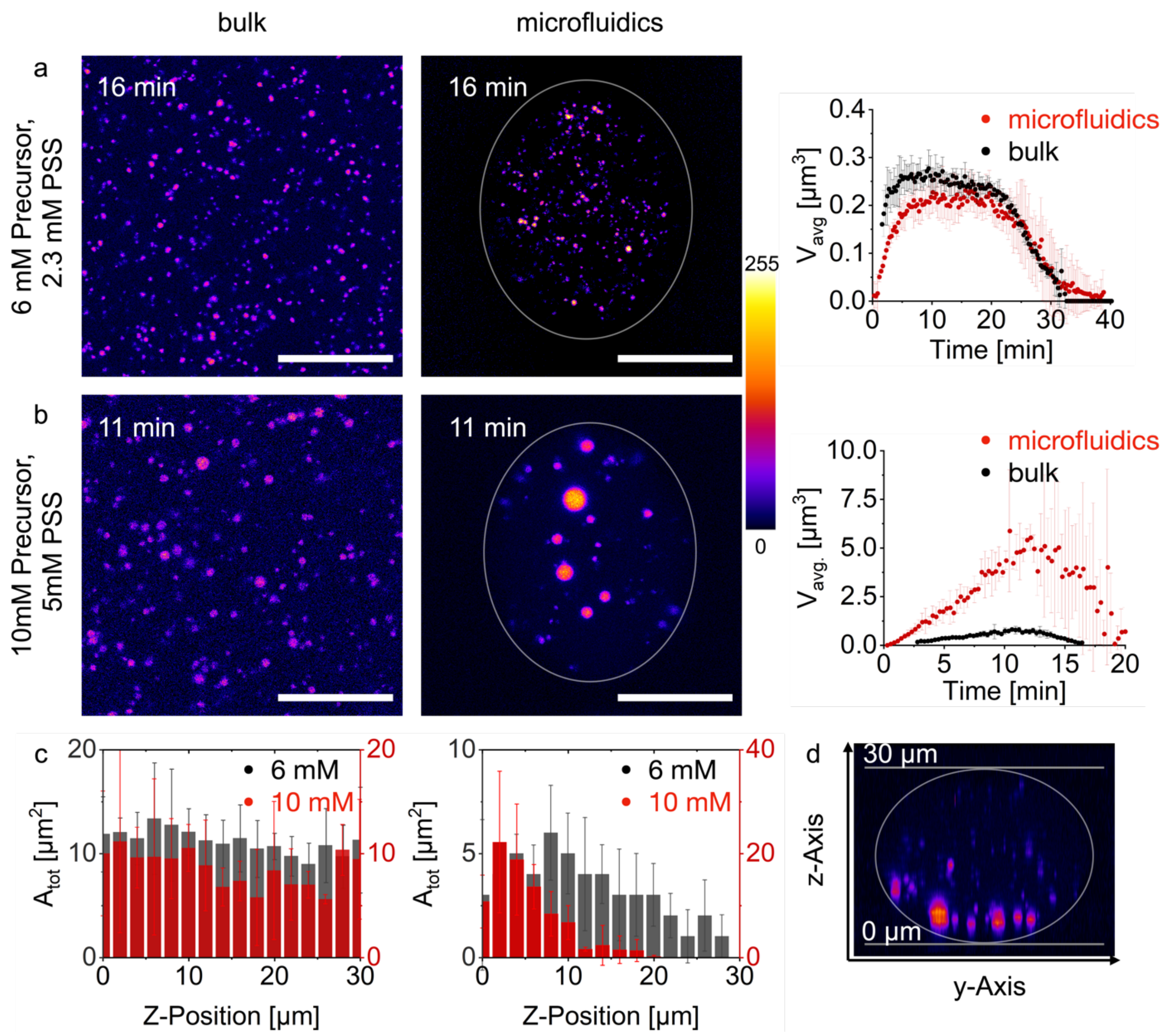

Supporting figure 4. Fusion of coacervates is influenced by the confinement. a *A comparison of the z-projection of coacervate-based droplets imaged in the bulk and imaged in the confinement of a microfluidic droplet. Both images are taken 16 min after the addition of 10 mM EDC. The pseudocolor-coded confocal image represents a maximum z-projection of a z-stack throughout one microfluidic droplet. The grey value scale from 0 to 255 is given next to the images. The scale bar represents 20 µm. A comparison of the quantitative analysis of both techniques shows that the average volume of the coacervate-based droplets is the same.* ***b*** *A comparison of the z-projection of coacervate-based droplets imaged in the bulk and imaged in the confinement of a microfluidic droplet. Both images are taken 11 min after the addition of 10 mM EDC. The scale bar represents 20 µm. A comparison of the quantitative analysis of both techniques shows that the average volume of the coacervate-based droplets in the microfluidic droplets is significantly bigger than the respective average volume in the bulk setup.* ***c*** *In the bulk setup the total area of imaged coacervate-based droplets is similar within a range of 30 µm in the direction of the z-position. In the microfluidic setup the total area of imaged coacervate-based droplets is only similar over a range of 30 µm in the z-direction for conditions under which coacervate-based droplets with an average volume of less than 0.3 µm^3 (6 mM precursor) are formed. For conditions with coacervate-based droplets of sizes up to 5-6 µm^3 the total area of imaged coacervate-based droplets is significantly higher towards the bottom of the microfluidic droplet.* ***d*** *z-y-projection of a microfluidic droplet containing 10 mM precursor and 5 mM pSS 11 min after EDC addition. Most coacervate-based droplets sit at the bottom of the microfluidic droplet.*

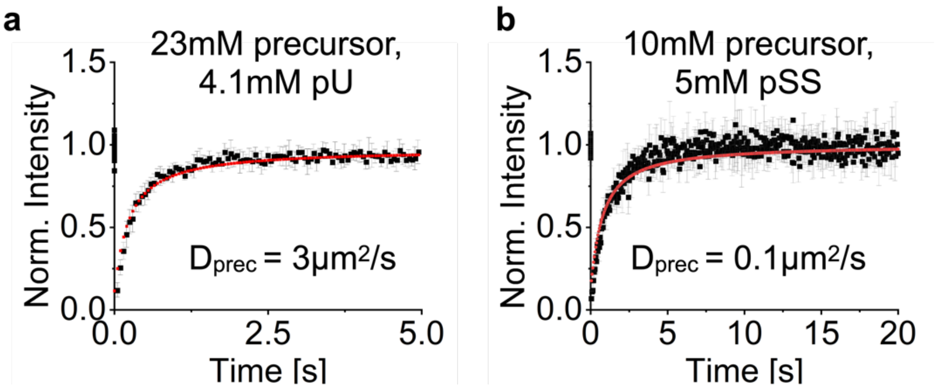

Supporting figure 5. Comparison of the precursor diffusivity inside pU- and pSS- based droplets. ***a*** *Fluorescence recovery after photobleaching of NBD-G(RG)$_3$D-OH in pU-based droplets.* ***b*** *Fluorescence recovery after photobleaching of NBD-G(RG)$_3$D-OH in pSS-based droplets. The red solid line represents the fit of the fluorescence recovery. Error bars represent standard deviation from 3 measurements.*

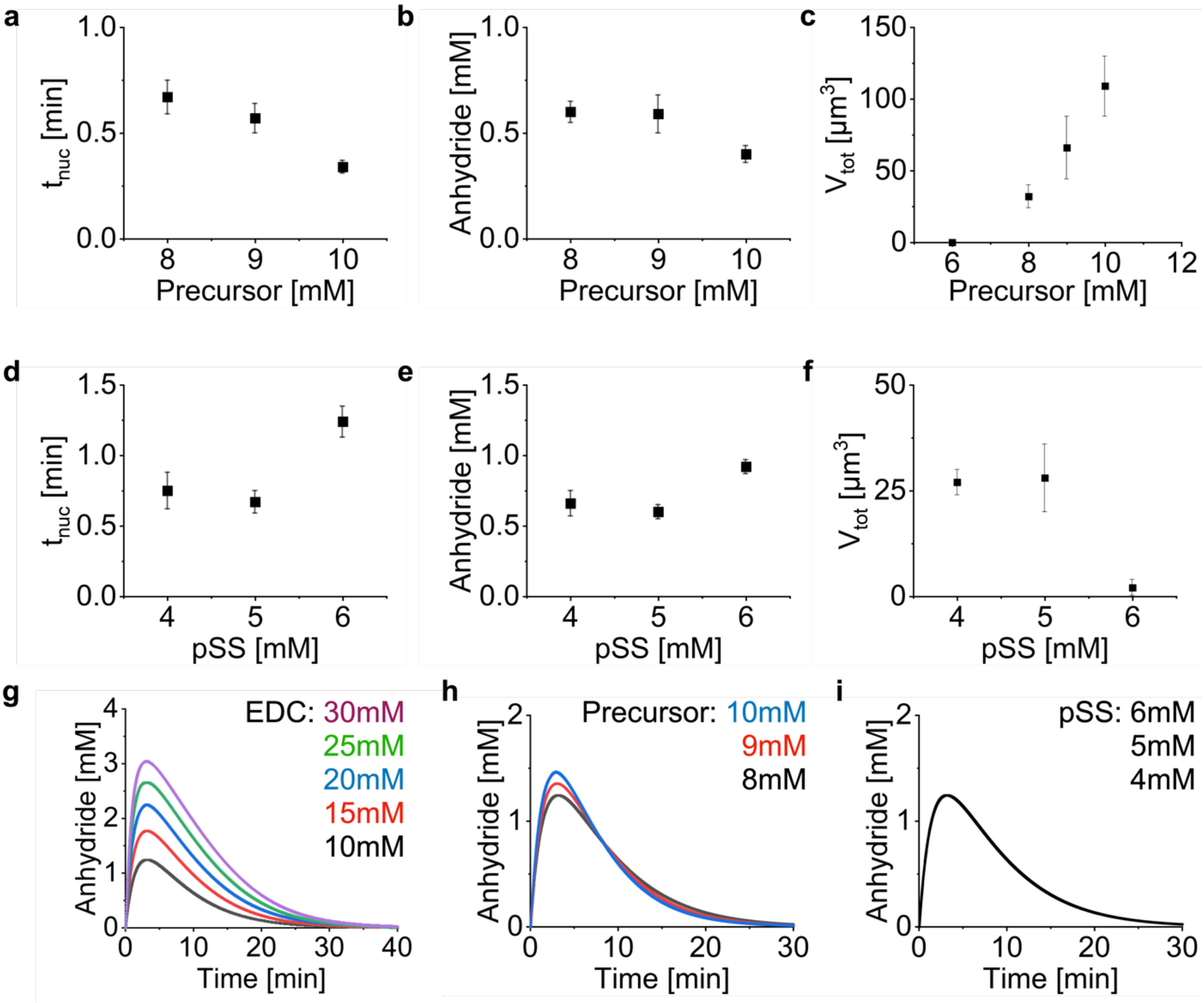

Supporting figure 6. Analysis of the nucleation time and the total volume of coacervate-based droplets. *Standard conditions in these experiments are 8 mM Ac-F(RG)₃D-OH, 5 mM pSS, 10 mM EDC, 0.1 µM sulforhodamine B in 200 mM MES at pH 5.3 with a variation in the concentration of the respective component.* ***a*** *Dependency of the nucleation time on the precursor concentration.* ***b*** *The anhydride concentration at the timepoint of nucleation dependent on the precursor concentration. Anhydride concentration is determined with the kinetic model at the time of nucleation.* ***c*** *Dependence of the total volume of coacervate-based droplets on the precursor concentration.* ***d*** *Dependence of the nucleation time on the pSS concentration.* ***e*** *The anhydride concentration at the timepoint of nucleation dependent on the pSS concentration. Anhydride concentration is determined with the kinetic model at the time of nucleation.* ***f*** *Dependence of the total volume of coacervate-based droplets on the pSS concentration.* ***g*** *Anhydride concentration for different initial EDC concentrations calculated with the kinetic model.* ***h*** *Anhydride concentration for different initial precursor concentrations calculated with the kinetic model.* ***i*** *Anhydride concentration for different initial pSS concentrations calculated with the kinetic model. Note that pSS is not part of the kinetic model.*

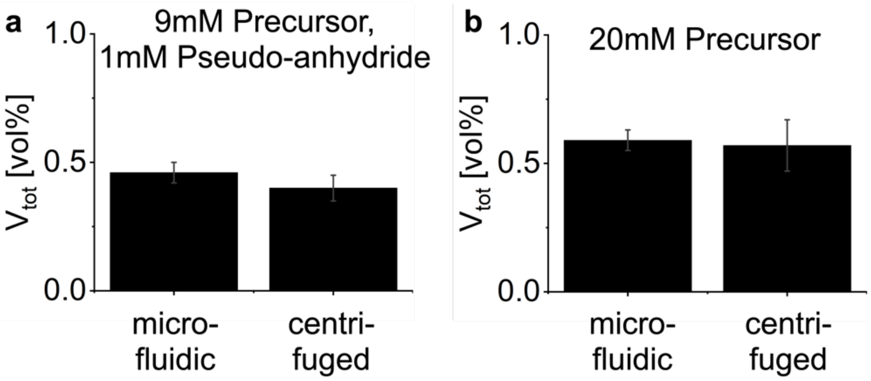

Supporting figure 7. Comparison of the total volume of coacervate-based droplets measured by centrifugation and in the microfluidic setup. *The total volume of non-fuel-driven coacervate-based droplets is measured by centrifugation and by confocal microscopy in the microfluidic setup.* ***a*** *9mM of the precursor Ac-F(RG)$_3$D-OH and 1mM of the pseudo-anhydride Ac-F(RG)$_3$N-NH$_2$ with 5 mM pSS in 200 mM MES at pH 5.3 were used to mimic the maximum anhydride level of the fuel-driven system with the conditions 10 mM Ac-F(RG)$_3$D-OH, 5 mM pSS, 10 mM EDC in 200 mM MES at pH 5.3. Error bars represent the standard deviation of 3 experiments.* ***b*** *20 mM Ac-F(RG)$_3$D-OH with 5 mM pSS in 200 mM MES at pH 5.3 were used as a second non-fuel-driven system. Under these conditions, coacervation takes place without the need for anhydride formation. Error bars represent the standard deviation of 3 experiments.*

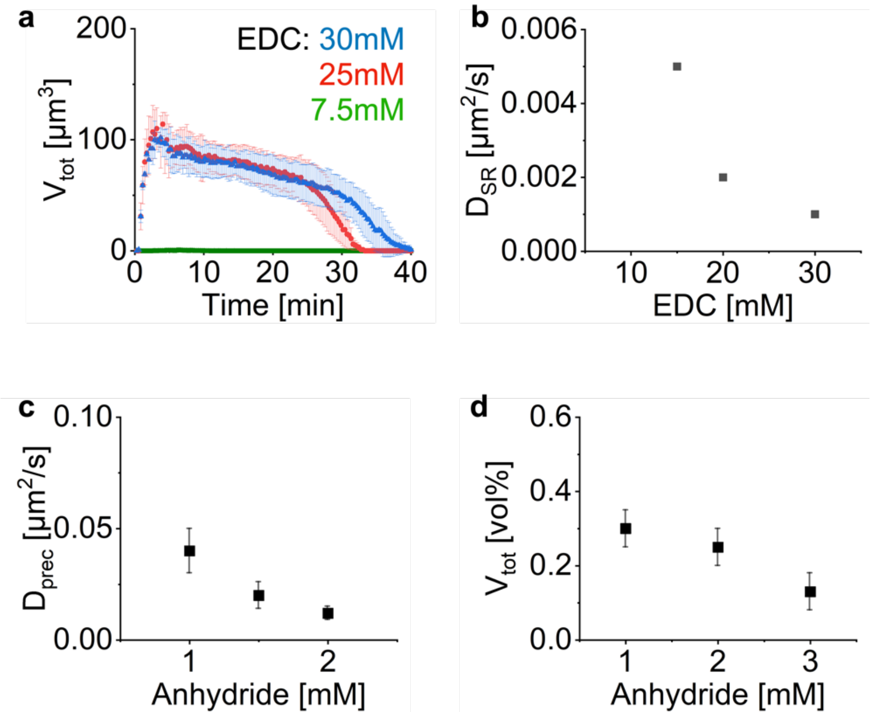

Supporting figure 8. Analysis of the total volume and viscosity of coacervate-based droplets. *Standard conditions in these experiments are 8 mM Ac-F(RG)$_3$D-OH, 5 mM pSS in 200 mM MES at pH 5.3 with a variation in the concentration of the respective component.* ***a*** *Analysis of the total volume of coacervate-based droplets over the entire reaction cycle depending on the fuel concentration. The total volume is given as volume percent defined as the total volume of coacervate-based droplets divided by the total volume of the microfluidic droplet. Error bars represent the standard deviation of 3 experiments.* ***b*** *The diffusivity of sulforhodamine B inside of coacervate-based droplets depending on the fuel concentration. The diffusivity is determined by FRAP experiments with 0.1 µM sulforhodamine B.* ***c*** *The diffusivity of NBD-G(RG)$_3$D-OH inside of coacervate-based droplets depending on the concentration of the pseudo anhydride Ac-F(RG)$_3$N-NH$_2$. The diffusivity is determined by FRAP experiments with 1 µM NBD-G(RG)$_3$D-OH.* ***d*** *Dependency of the total volume of coacervate-based droplets on the concentration of the pseudo-anhydride Ac-F(RG)$_3$N-NH$_2$. The volume of the coacervate-based droplets is determined by centrifugation. Error bars represent the standard deviation of 3 experiments.*

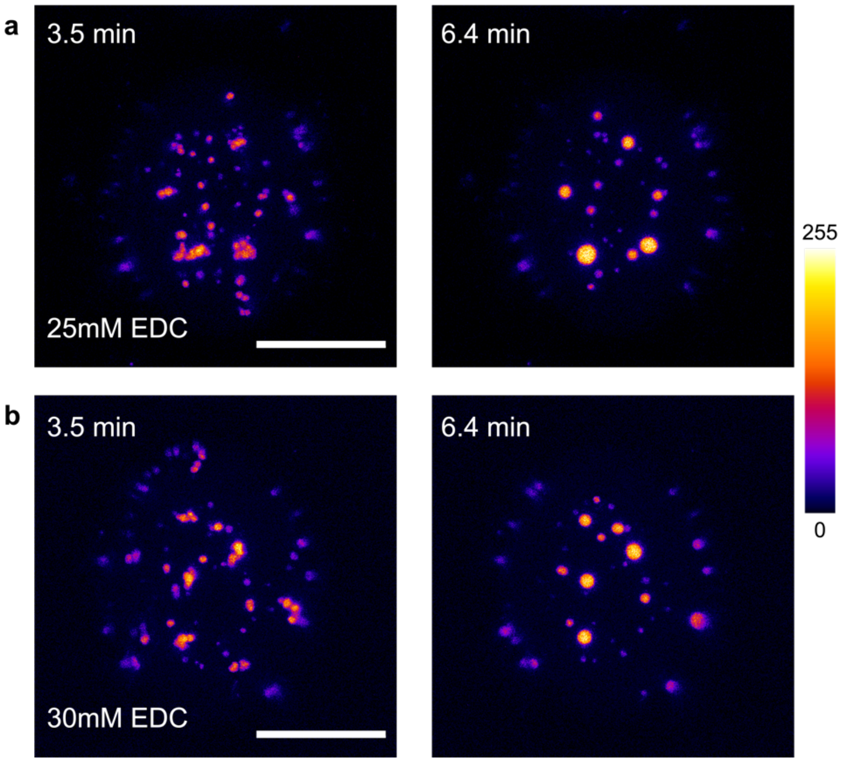

Supporting figure 9. Agglomerate formation. *Standard conditions in these experiments are 8 mM Ac-F(RG)$_3$D-OH, 5 mM pSS, 0.1 µM sulforhodamine B in 200 mM MES at pH 5.3 with the respective EDC concentration.* ***a-b*** *Coacervate-based droplets initially form agglomerate-like structures after fusion. After several minutes the spherical shape is restored. Images are z-projections at the given time point. Scale bars represent 20 µm.*

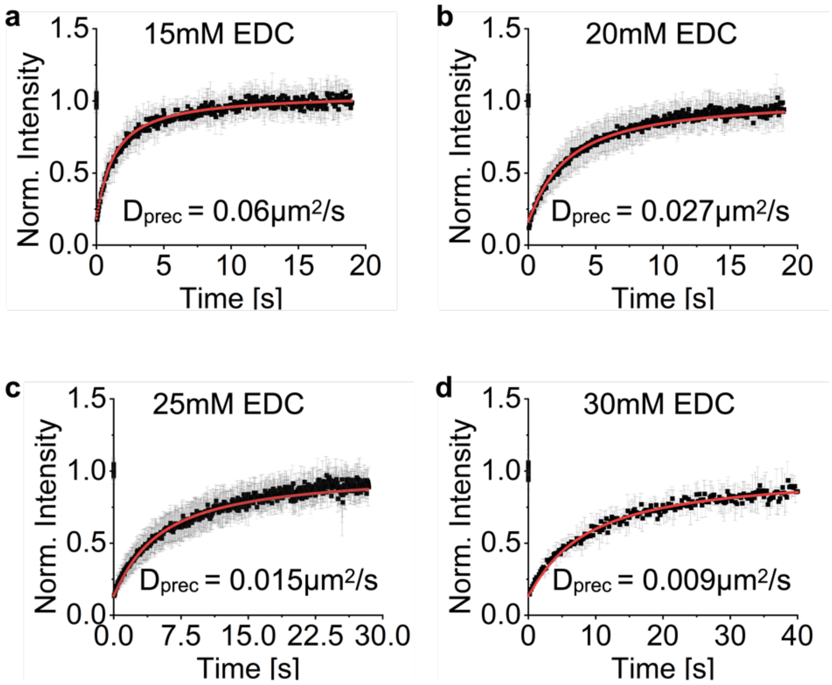

Supporting figure 10. Comparison of precursor diffusivity inside pSS-based droplets depending on the EDC concentration. *For all experiments, 8 mM Ac-F(RG)$_3$D-OH, 5 mM pSS, and 1μM NBD-G(RG)$_3$D-OH in 200 mM MES at pH 5.3 was used.* ***a-d*** *Fluorescence recovery after photobleaching of NBD-G(RG)$_3$D-OH in coacervate-based droplets depending on the fuel concentration. The red solid line represents the fit of the fluorescence recovery. Error bars represent standard deviation from 9 measurements.*

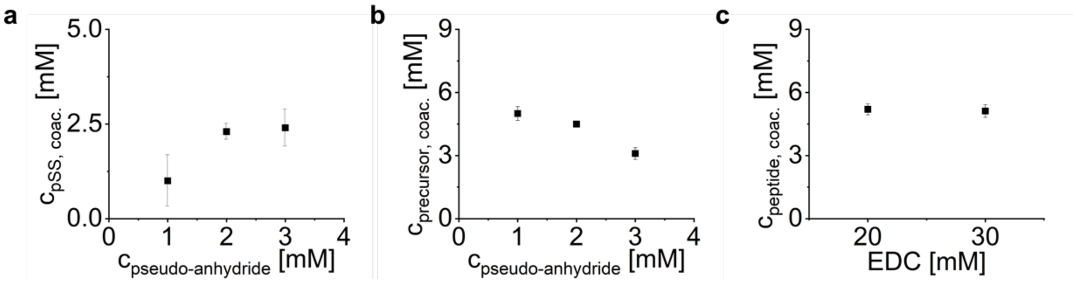

Supporting figure 11. Fluorescence partitioning of precursor, pSS, and the total amount of peptide in coacervate-based droplets. a *Fluorescence partitioning of 1 µM Cy-5 pSS in non-fuel-driven coacervate-based droplets depending on the concentration of pseudo-anhydride. The amount of pSS that is missing in the supernatant after centrifugation is regarded as the amount of pSS that partitioned into the coacervate-based droplets. Combined concentrations of Ac-F(RG)$_3$D-OH and Ac-F(RG)$_3$N-NH$_2$ are 8 mM with 5 mM pSS in 200 mM MES at pH 5.3. Error bars represent standard deviations from 3 measurements.* ***b*** *Fluorescence partitioning of 1 µM NBD-G(RG)$_3$D-OH in non-fuel-driven coacervate-based droplets depending on the concentration of pseudo-anhydride. The amount of precursor that is missing in the supernatant after centrifugation is regarded as the amount of precursor that partitioned into the coacervate-based droplets. In the non-fuel-driven case NBD-G(RG)$_3$D-OH is only a measure for the precursor since no activation to the anhydride is possible. Combined concentrations of Ac-F(RG)$_3$D-OH and Ac-F(RG)$_3$N-NH$_2$ are 8 mM with 5mM pSS in 200 mM MES at pH 5.3. Error bars represent standard deviations from 3 measurements.* ***c*** *Fluorescence partitioning of 1 µM NBD-G(RG)$_3$D-OH in fuel-driven coacervate-based droplets depending on the concentration of EDC. The amount of peptide that is missing in the supernatant after centrifugation is regarded as the amount of peptide that partitioned into the coacervate-based droplets. In the fuel-driven case, NBD-G(RG)$_3$D-OH is considered as a measure for both the precursor as well as the anhydride since it is able to form the anhydride itself. Combined concentrations of Ac-F(RG)$_3$D-OH and Ac-F(RG)$_3$N-NH$_2$ are 8 mM with 5mM pSS in 200 mM MES at pH 5.3. Error bars represent standard deviation from 3 measurements.*

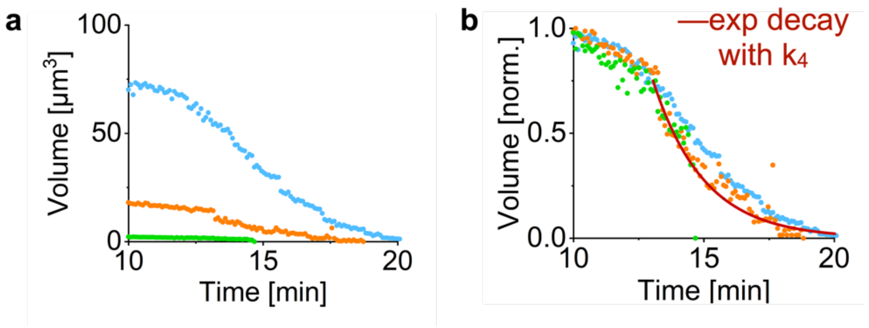

Supporting figure 12. ***Comparison of the volume decay. a*** *Volume decay profile of the blue, orange, and green droplets from figures 5a and c.* ***b*** *Normalization of the volume decay shows that all three droplets decay with a similar rate constant. The red solid line represents an exponential decay using the hydrolysis constant k_4 from the kinetic model as the constant k for exponential volume decay with the formula $y = V_0 e^{-kt}$.*

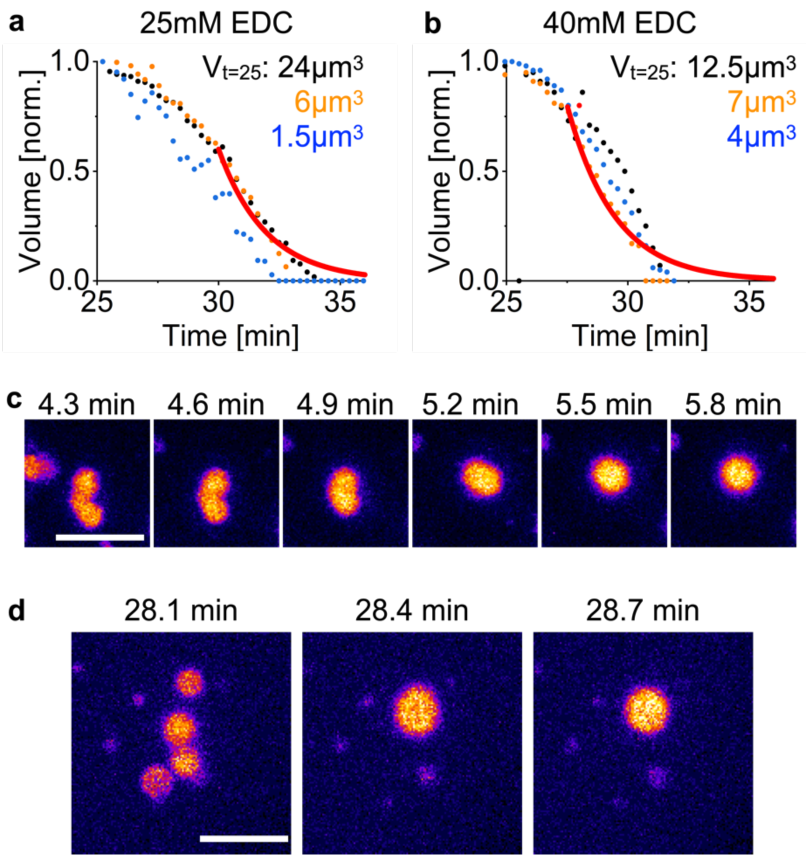

Supporting figure 13. ***Comparison of the volume decay for high fuel concentrations.*** *For all experiments 8 mM Ac-F(RG)$_3$D-OH, 5 mM pSS and 0.1 µM sulforhodamine B in 200 mM MES at pH 5.3 were used.* ***a,b*** *Normalization of the volume decay for three droplets of different starting volumes shows that all three droplets decay with a similar rate constant. The red solid line represents an exponential decay using the hydrolysis constant k_4 from the kinetic model as the constant k for exponential volume decay with the formula $y = V_0 e^{-kt}$. The coacervate-based droplets were fueled with the indicated amount.* ***c*** *Time series of a fusion early in the reaction cycle with a high ratio of activated to deactivated species present. The scale bar represents 5µm.* ***d*** *Time series of a fusion event late in the reaction cycle with a low ratio of activated to deactivated species present. The scale bar represents 5µm. c and d show coacervate-based droplets from the same experiment in which they were fueled with 40 mM EDC.*

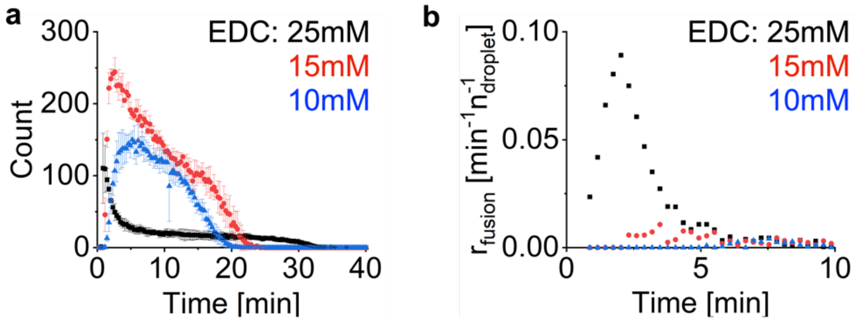

Supporting figure 14. ***Comparison of the droplet number and the average droplet fusion rate.*** *For all experiments 8 mM Ac-F(RG)$_3$D-OH, 5 mM pSS and 0.1 µM sulforhodamine B in 200 mM MES at pH 5.3 were used with varying EDC concentrations.* ***a*** *The number of coacervate-based droplets that were detected at each time point during the reaction cycle above a radius threshold of 400 nm.* ***b****. The average likelihood of every coacervate-based droplet to undergo a fusion event. The rate of fusion is defined as the decline in droplet number in comparison to the previously measured data point. Fusion rates are only calculated after the maximum number of coacervate-based droplets has been reached and until the average size of coacervate-based droplets declines. The decline in the average size is used as an indicator for the start of droplet dissolution. The fusion rates are plotted with a rolling average of n=5.*

Refrences

[1] a) F. Spath, C. Donau, A. M. Bergmann, M. Kranzlein, C. V. Synatschke, B. Rieger, J. Boekhoven, *J Am Chem Soc* **2021**, *143*, 4782-4789; b) F. Schnitter, A. M. Bergmann, B. Winkeljann, J. Rodon Fores, O. Lieleg, J. Boekhoven, *Nat Protoc* **2021**, *16*, 3901-3932.

[2] D. C. Duffy, J. C. McDonald, O. J. A. Schueller, G. M. Whitesides, *Analytical Chemistry* **1998**, *70*, 4974-4984.

[3] a) M. Weiss, J. P. Frohnmayer, L. T. Benk, B. Haller, J. W. Janiesch, T. Heitkamp, M. Borsch, R. B. Lira, R. Dimova, R. Lipowsky, E. Bodenschatz, J. C. Baret, T. Vidakovic-Koch, K. Sundmacher, I. Platzman, J. P. Spatz, *Nat Mater* **2018**, *17*, 89-96; b) T. W. Hofmann, S. Hanselmann, J. W. Janiesch, A. Rademacher, C. H. Bohm, *Lab Chip* **2012**, *12*, 916-922.

[4] C. Wu, C. D. Allis, *Chromatin and Chromatin Remodeling Enzymes, Part A*, Elsevier Science, **2004**.

[5] A. B. Kayitmazer, H. B. Bohidar, K. W. Mattison, A. Bose, J. Sarkar, A. Hashidzume, P. S. Russo, W. Jaeger, P. L. Dubin, *Soft Matter* **2007**, *3*, 1064-1076.

[6] W.-H. Tsai, *Computer Vision, Graphics, and Image Processing* **1985**, *29*, 377-393.

[7] J. Y. Tinevez, N. Perry, J. Schindelin, G. M. Hoopes, G. D. Reynolds, E. Laplantine, S. Y. Bednarek, S. L. Shorte, K. W. Eliceiri, *Methods* **2017**, *115*, 80-90.
